# Finite element analysis of primary healing implants with different transmucosal designs

**DOI:** 10.1111/jopr.14044

**Published:** 2025-03-03

**Authors:** Mario Ceddia, Tea Romasco, Luca Comuzzi, Alessandro Cipollina, Adriano Piattelli, Natalia Di Pietro, Bartolomeo Trentadue

**Affiliations:** ^1^ Department of Mechanics, Mathematics and Management Polytechnic University of Bari Bari Italy; ^2^ Department of Medical, Oral and Biotechnological Sciences “G. D'Annunzio” University of Chieti‐Pescara Chieti Italy; ^3^ Center for Advanced Studies and Technology (CAST), “G. D'Annunzio” University of Chieti‐Pescara Chieti Italy; ^4^ Division of Dental Research Administration Tufts University School of Dental Medicine Boston Massachusetts USA; ^5^ Independent Researcher San Vendemiano Italy; ^6^ Independent Researcher Sciacca Italy; ^7^ School of Dentistry Saint Camillus International University of Health and Medical Sciences Rome Italy; ^8^ Facultad de Medicina UCAM Universidad Católica San Antonio de Murcia Murcia Spain; ^9^ Present address: Division of Dental Research Administration Tufts University School of Dental Medicine Boston MA USA

**Keywords:** conometric connection, dental implants, Finite element analysis, Finite element method, osseointegration, primary healing implant, stress, transmucosal implant abutments

## Abstract

**Purpose:**

This study aimed to assess the response of peri‐implant tissues, both hard and soft, to mechanical stress when using a primary healing implant (PHI) with two different transmucosal profiles: concave (Model A) and divergent (Model B). The investigation also sought to observe bone modeling under post‐extraction conditions.

**Materials and Methods:**

The methodology involved the creation of a three‐dimensional bone model of the first molar region, derived from a computed tomography scan. Subsequently, two implants were inserted into the bone site and subjected to a loading force of 100 N at a 45° angle.

**Results:**

The results of stress analysis, using the von Mises criterion, indicated that Model A exhibited a more uniform stress distribution within the soft tissues, registering a maximum value of 75 MPa, in contrast to 126 MPa observed in Model B. Concerning implant stress, the peak value was recorded at the conometric connection zone between the implant and the abutment, measuring 138 MPa for Model B and 125 MPa for Model A. The study specifically analyzed cortical bone stress, which revealed levels of 72 MPa for Model B and 64 MPa for Model A. Additionally, stress distribution in immature bone ranged from 1.3 to 9 MPa for Model A and from 1.5 to 12 MPa for Model B.

**Conclusions:**

The finite element method represents a valuable tool for the design and optimization of implant shapes, taking into account occlusal loads and specific anatomical locations. This approach aims to enhance the stimulation of both soft and hard tissues, thereby mitigating the risk of implant failure.

The process of resorption following tooth extraction has been extensively documented, resulting in a reduction in the buccolingual and apicocoronal dimensions of the alveolar ridge.^1^, ^2^, ^3^, ^4^, ^5^, ^6^, ^7^, ^8^, ^9^, ^10^, ^11^ The implementation of immediately loaded dental implants provides a long‐term solution to this matter by replacing the tooth root and offering support to the crown during functional activities.^12^, ^13^, ^14^ Extensive scientific research validates the long‐term success of osseointegrated implants, in accordance with the biological principles initially established by Brånemark.^15^ Brånemark's original protocol advocates for complete healing of the alveolar bone subsequent to tooth extraction, before the placement of a dental implant, a process which typically spans 6 to 12 months. Throughout this period, it has been observed that there is a potential loss of 44% or more of the alveolar ridge due to bone resorption.^16^, ^17^, ^18^

On the other hand, in a study by Schenk et al.,^19^ the concept of primary healing was examined, suggesting that implants inserted in bone with precise coupling between fixture and implant site preparation, without stressing the bone, can achieve healing outcomes comparable to those following Brånemark's protocol. Piattelli et al.^20^ documented the benefits of immediate implant placement post‐tooth extraction, such as preservation of the extraction site, enhanced secondary stability leveraging granulation tissue healing directly around the implant, and simplification of surgical procedures. This immediate implant placement, referred to as primary healing implant (PHI),^21^ has demonstrated high long‐term success rates exceeding 90% in clinical studies, with notable bone‐to‐implant contact (BIC).^22^, ^23^, ^24^, ^25^ Furthermore, Paolantonio et al.^25^ reported that histomorphometric analysis revealed no significant differences in the percentage of direct bone contact between conventional implants and PHI implants. Thus, implant placement directly into healing bone appears to be a viable and safe approach, yielding results comparable to traditional implants inserted in healed bone.

The success of dental implant treatment depends on the stability provided by both the surrounding hard and soft tissue. Various factors, including implant loading, tissue management, and regular follow‐up, play a pivotal role in the outcome of the treatment.^26^, ^27^ Ensuring the presence of adequate soft and hard tissue is imperative for the success of the procedure. A functional implant must establish a biological connection with living tissue by penetrating the oral mucosa, which serves as a protective barrier for the underlying bone structures.^28^, ^29^ Additionally, the presence of a sufficient amount of keratinized gingiva is essential for improved implant stabilization. Consequently, the configuration of the transmucosal zone of the implant holds significant importance in maintaining the biological barrier. A study conducted by Valente et al.^30^ revealed that implants with convergent or concave transmucosal profiles exhibited a positive influence on soft tissue, particularly in terms of marginal bone loss (MBL) and peri‐implant soft tissue aesthetics, as compared to implants with parallel or divergent profiles. Moreover, converging abutment profiles, irrespective of their surface micro‐geometry, were found to promote axial development of peri‐implant connective tissue.^31^, ^32^, ^33^ Stress significantly affects the biomechanical properties of oral soft tissues. It has the potential to alter the viscoelastic response of tissues, subsequently impacting their ability to dissipate energy during loading and return to their original shape post‐removal of the load.^34^ Increased mechanical stress can lead to an elevation in interstitial pressure, potentially exceeding vascular pressure.^34^ This phenomenon may result in a reduction of blood flow to the affected tissue, thereby inducing ischemia and, in extreme cases, tissue necrosis. Furthermore, mechanical stress can modify the structural integrity of collagen and the extracellular matrix within soft tissues. Such alterations can impact the biomechanical properties of the tissue, rendering it more vulnerable to injury and impairing its overall functionality.

Therefore, it is essential to understand the distribution of stress in hard and soft tissues to prevent potential issues such as bone loss, implant failure, or damage to surrounding structures.^34^, ^35^, ^36^ Finite element analysis (FEA) is a numerical technique used to assess stresses in complex structures under external loads. In the context of dental implants, FEA can evaluate stress distribution on the implant structure and surrounding tissues under conditions closely resembling reality, contributing to more effective and safer solutions in dental implantology.^37^, ^38^, ^39^, ^40^, ^41^ This study aimed to use FEA to assess the impact of PHIs with two different abutment shapes (concave: Model A, and divergent: Model B) on stress transmission in hard and soft tissues after tooth extraction. By modeling the mechanical behavior of the gingiva, often overlooked in many FEA studies, this research will enhance understanding of how soft and hard tissues respond to mechanical loading.

The novelty of this study, in comparison to clinical studies, lies in the application of the finite element method (FEM). This approach facilitates the examination of stress and strain distributions within both soft and hard tissues, thereby enabling the identification of regions that may potentially become critical. Considering the implant placement technique, the current study will examine whether post‐extraction bone or granulation tissue behaves differently around the two abutment configurations (null hypothesis).

## MATERIALS AND METHODS

### Modeling

**FIGURE 1:**
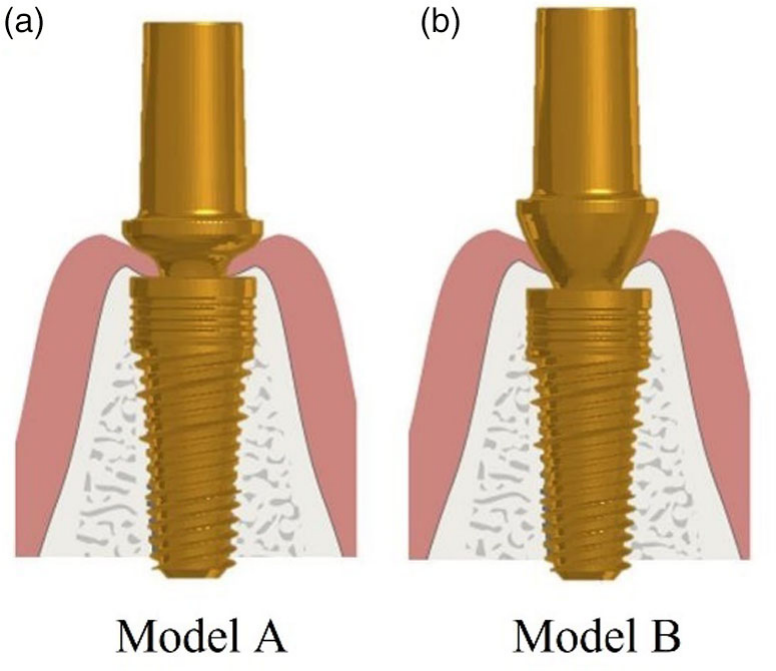
Abutment designs. (a) Concave (Model A) and (b) divergent (Model B).

In the present investigation, an analysis was conducted on a PHI (EVO MAC, Lancer Global Srl, Trezzano, Italy) characterized by a 5 mm diameter and 8 mm length, featuring a through screw, a 6 conometric connection, and a switching platform.^42^ The surface of these implants was treated through an initial angular quartz sandblasting process (grit 220) combined with the double acid etching (DAE) treatment technique. The titanium implants and final abutments were coated with titanium nitride (TiN) via the physical vapor deposition (PVD) process. The gold color of the TiN coating serves as an aesthetic barrier, preventing the implant from being visible through the mucosal tissue in backlighting, repelling negative bacterial load, and enhancing osseointegration.^43^, ^44^ The morphology of the PHI implant is characterized by its cylindrical body in the coronal part and conical in the medullary part, large self‐centering coils with a 1.5 mm pitch, and osteogenic corrugations. Additionally, the implant features a three‐principle apical coil with a 0.5 mm pitch, which contributes to promoting primary stability. This study evaluated two distinct abutments with different profiles: concave (Model A) and divergent (Model B) (Figure 1).

**FIGURE 2:**
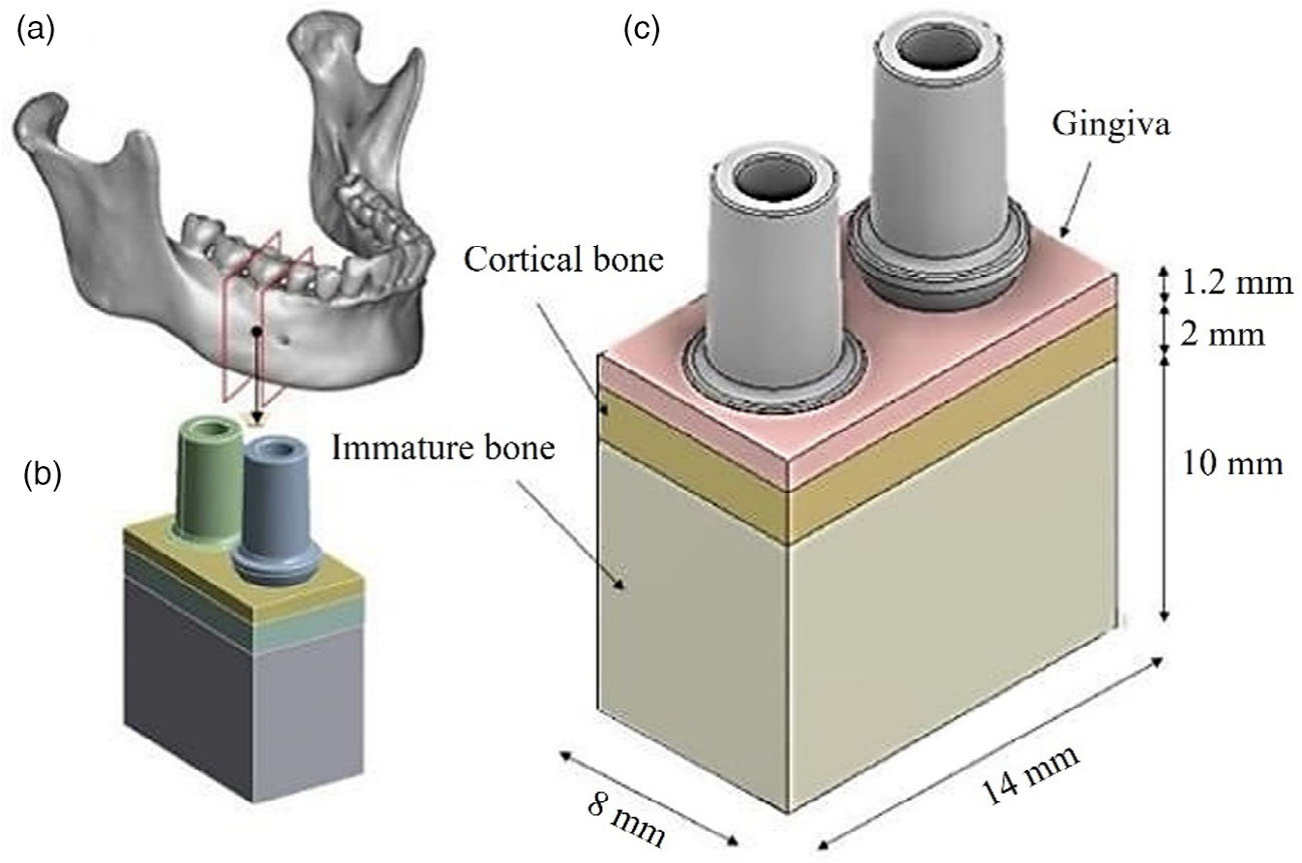
Modeling of the implants along with the soft (gingiva) and hard (cortical and spongiosa) tissues. (a) A section of the bone generated from a CT scan^45^; (b) the 3D cross‐section of the mandible created using Autodesk Inventor 2023 (San Francisco, CA, USA); (c) the comprehensive model encompassing the various dimensions, with Model A implant on the left and Model B implant on the right. 3D. three‐dimensional; CT, computed tomography.

Once the implant's geometry was established, the soft tissue and hard tissue models were defined. Specifically, a three‐dimensional (3D) bone block (hard tissue) model was reconstructed and designed using computer‐aided design (CAD) software (Autodesk Inventor 2023, San Francisco, CA, USA) based on a computed tomography (CT) scan of a patient, as documented in previous studies^45^, ^46^ (Figure 2a, b). A cross‐sectional image of the right first molar region with a width of 14 mm and a vertical height of 13.2 mm was utilized for this study (Figure 2c). The thickness of the cortical bone utilized in this study was established at 2 mm, which aligns with the D2 classification bone type according to the Misch classification system. For the modeling of the gingival component (soft tissue), a thickness of 1.2 mm was selected at the interface with the lower surface of the abutment, intentionally excluding the unattached portion of the gingiva adjacent to the abutment.^47^ The portion of the gingiva that does not contact the abutment was not considered to affect the distribution of stress.

Subsequently, the bone model was used to create two cylindrical cavities with a diameter of 4.3 mm, following the manufacturer's indications for creating an undersized osteotomy. Implants were then inserted subcrestally to a depth of 0.5 mm (Figure 2). Then, the mirror image of the implant threads was created using the solid edit command in Autodesk Inventor 2023, to assume complete contact between the bone and the implant.

### Materials

In relation to bone, its characteristics were defined in accordance with Misch's proposed bone quality classification.^48^, ^49^ The right first molar region was selected as the anatomical focus of this investigation. A D2 classification and a measured density of 1250 Hounsfield units (HU) were employed for this study. When quantifying the mechanical properties of bone, the relationship between stiffness (E) and density is often expressed as 𝐸∝𝜌𝛼. For cortical bone, 𝛼 can range between 4–7.4, and for trabecular bone, it can vary between 1.27–2.57.^50^, ^51^, ^52^ It is important to note that in the post‐extraction scenario, the bone undergoes fracturing, and during the healing process, it transitions from an initial weaker and less rigid tissue, known as callus, which primarily consists of connective tissue, cartilage, and immature bone, to more mature and resistant bone tissue. This bone remodeling process leads to an increase in the strength and stiffness of the healed bone compared to the initial healing phase.^53^ Table 1 provides an overview of the main tissues formed during the bone healing process and their properties.

**TABLE 1:** Elastic properties of modeled bones.^53^, ^54^

Material	Young's modulus, E (GPa)	Poisson coefficient, ν
Cortical bone	17	0.30
Immature bone (E_I_)	1	0.30
Cartilage	0.01	0.47
Connective tissue	0.002	0.47
Granulation tissue (E_g_)	0.2	0.47

Abbreviations: E_I_, Young's modulus of immature bone; E_g_, Young's modulus of granulation tissue.

The determination of the local Young's modulus of post‐extraction healing bone (E_H_) is based on the mixing rule (Equation 1) derived from the Young's modulus of immature bone (E_I_) and granulation tissue (E_G_), as presented in Table 1.

(1) $$E_{H}=\frac{E_{g}\;E_{I}}{E_{g}+E_{I}}=0.16\;GPa$$

Conversely, the elastic modulus (E) of the gingiva is influenced by various factors, including the presence of keratin in the tissue, the organization of collagen fibers in the connective tissue, and the density and arrangement of elastic fibers.^53^ Specifically, keratinized gingiva exhibits higher stiffness and strength attributable to the presence of keratin in its stratum corneum and a greater concentration of collagen fibers in the connective tissue compared to the buccal mucosa, which has a higher proportion of epithelial elements and lower tensile strength. Consequently, the study considered keratinized gingiva while using grade 5 titanium alloy (Ti6Al4V) for the implant.^54^ The Young's modulus (E) and Poisson's ratio (ν) for the implant and abutment (Ti6Al4V) were 110 GPa and 0.3, respectively. For the keratinized gingiva, these values were 0.0197 GPa and 0.3, respectively.^53^, ^54^, ^55^ The gingiva, as a component of oral tissue, exhibits viscoelastic and hyperelastic characteristics. This implies that its mechanical response is influenced not only by the immediate deformation but also by temporal factors and loading history. However, an isotropic approach−where the mechanical properties are consistent in all directions−may be employed to streamline models and facilitate simulations. This assumption is reasonable given the limited number of numerical studies that have characterized this type of tissue.^53^, ^54^ Therefore, the materials simulated in this study were assumed to exhibit linear elastic, homogeneous, and isotropic behavior.

### Finite element analysis

The CAD file containing the 3D model in STP format was imported into the ANSYS Workbench 2023 software (R23^®^, Canonsburg, PA, USA). The model was primarily discretized with 10‐node tetrahedral elements (SOLID 187) with a maximum element size of 0.5 mm. Through sensitivity tests (convergence analysis), it was determined that an element size of less than 0.3 mm was suitable for accurately modeling the bone‐implant interface.^56^ This testing procedure facilitates the selection of the optimal mesh by identifying conditions under which the results demonstrated convergence. This is characterized by diminishing variations in the results as the mesh size is progressively reduced. Enhanced analysis of the results was achieved by using a mesh size of 0.3 mm at the bone‐implant contact. Furthermore, a frictional contact between the implant and abutment, as well as between the abutment and the screw, was assumed with a friction coefficient of 0.3.

The simulation anticipated the dissipation of force in multiple directions within the lower section of the bone block due to the imposed constraint conditions. To simulate the masticatory load during chewing on the first molar, an oblique force of 45° with an intensity of 100 N, relative to the implant's axis of symmetry, was applied to both abutments.^57^

The study considered bone‐to‐bone and gingiva‐to‐bone contact conditions, as well as implant‐bone contact, to be fixed. All models were analyzed using Windows 10 64‐bit with an Intel I7 processor and 16 GB of RAM. The numerical results were then translated into visual representations using color maps that ranged from blue (indicating the least stressed areas) to red (indicating the most stressed areas). Stress was determined based on the von Mises criterion. The analysis, conducted using ANSYS Workbench 2023 software (R23^®^, Canonsburg, PA, USA), was of a static nature, assuming constant loads over time.

## RESULTS

### Stress distribution on implants

**FIGURE 3:**
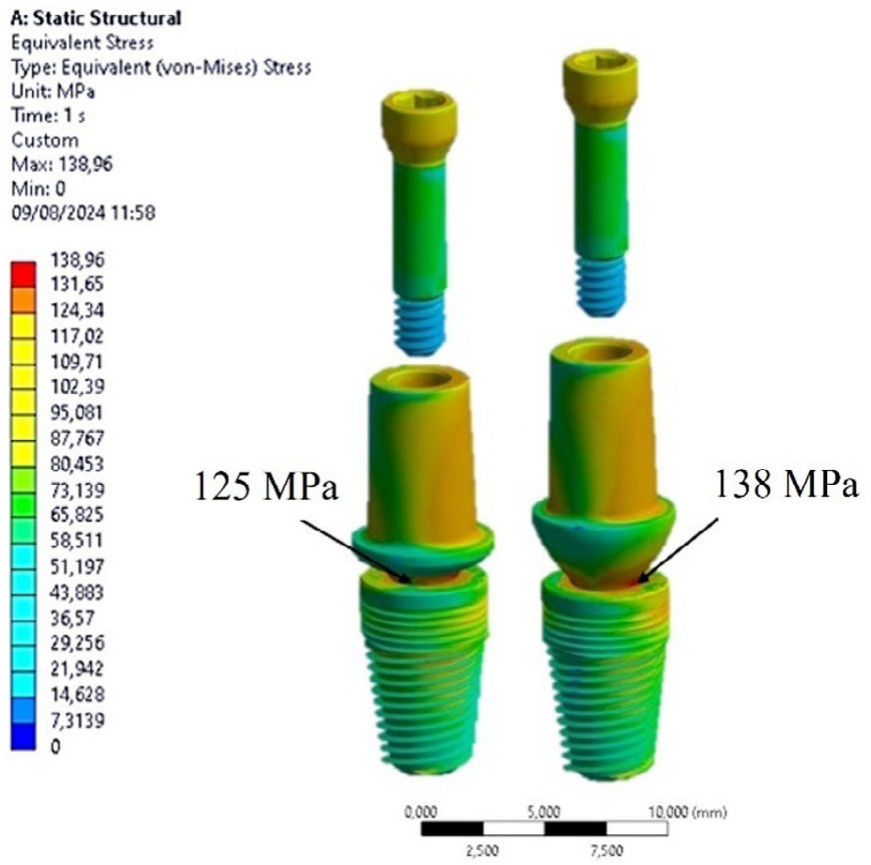
Maximum von Mises stress values for the implant and fixation screw. Left: Model A; right: model B.

The analysis revealed that both implants experienced maximum stresses under oblique loading, resulting in the generation of bending stresses at the implant‐abutment contact. Specifically, Figure 3 highlights that in the left configuration (Model A), the stress measured at the abutment‐implant contact was 125 MPa, whereas in the right configuration (Model B), the stress measured in the same area was 138 MPa. This demonstrates the significant influence of the abutment profile on stress transmission within the implant.

**FIGURE 4:**
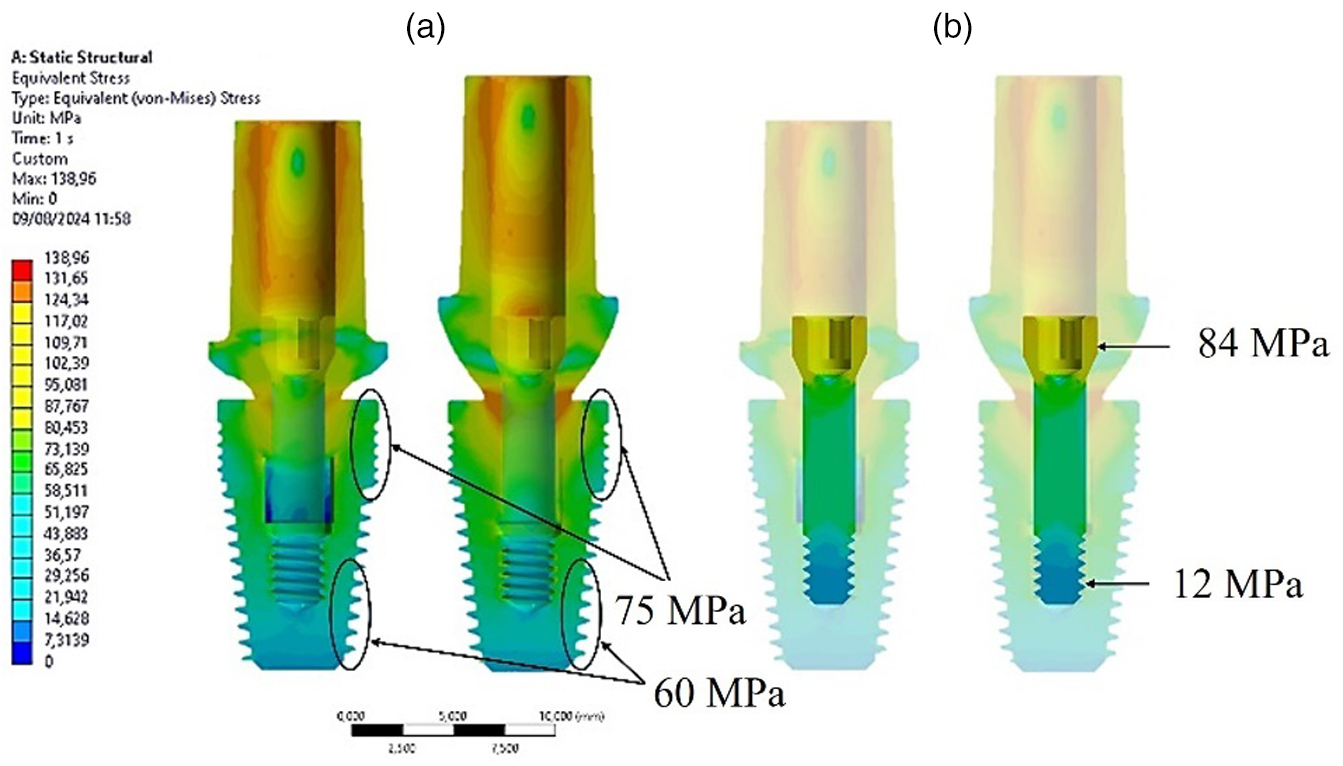
Cross‐sectional view of the von Mises stress distribution for the two implants. (a) On the implants; (b) on the abutment screws.

The cross‐sectional view depicted in Figure 4a illustrates the uniform stress transfer between the abutment and the implant at the conometric connection interface. Additionally, it is evident that the upper region of the implant, specifically at the implant neck and the first four threads, experienced stress of 75 MPa. Conversely, the apical area of the implant bore about 60 MPa stress in both implants.

The anchorage support provided by the retention screw between the abutment and the implant was substantial. Figure 4b indicates that the stress distribution is comparable in both Model A and Model B, with a maximum stress of approximately 84 MPa in the abutment connection zone and a lower stress of 12 MPa in the threaded zone.

These preliminary findings underscored the pivotal role of the abutment in transmitting stress to the prosthetic components. However, the tapered connection resulted in heightened stress transmission at the interface through the contact points. Additionally, the application of oblique loading accentuated the stress concentration at the implant‐abutment contact, specifically in Model B of the abutment.

### Stress distribution on bone

**FIGURE 5:**
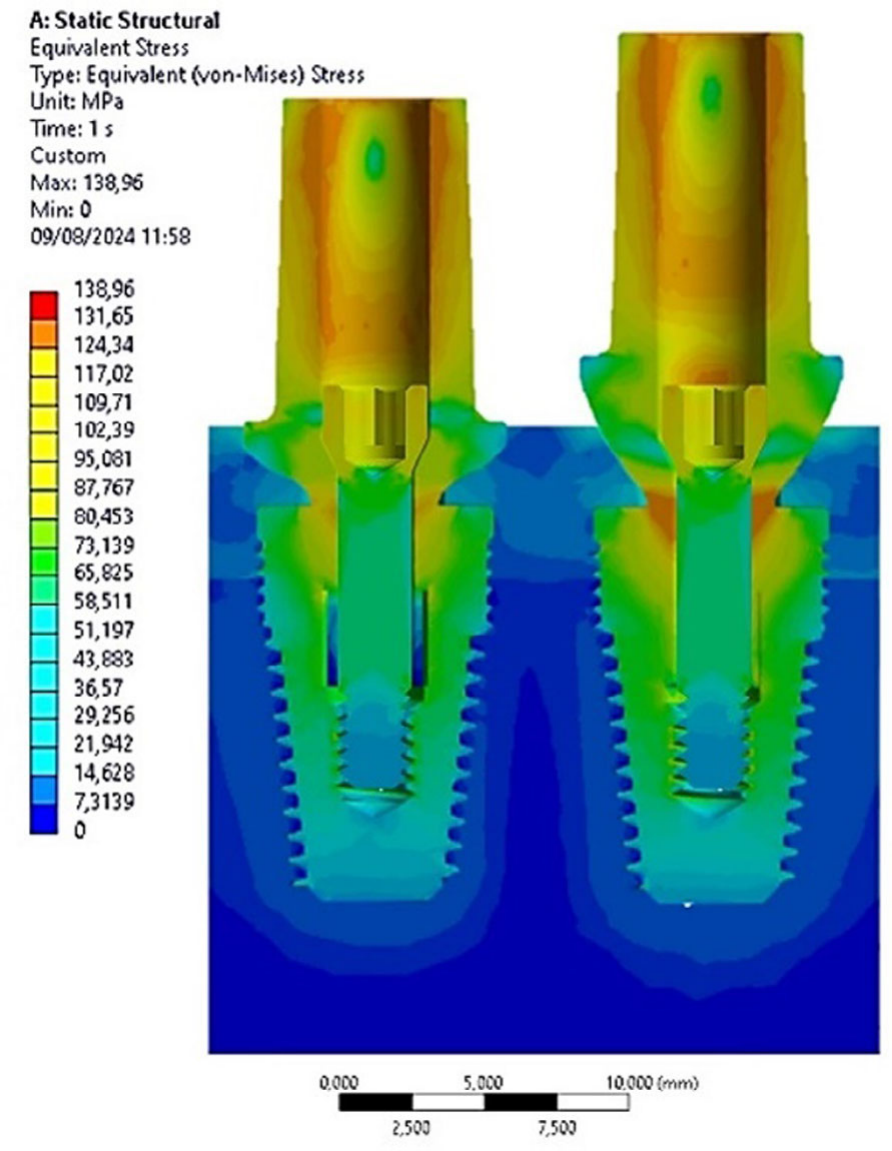
Stress distribution on the 3D model. Left plant (model A), right (model B). 3D, three‐dimensional.

The distribution of von Mises stresses in hard (cortical and healing bones) and soft (gingiva) tissues resulting from prosthetic components is illustrated in Figure 5. A notable observation is the occurrence of stress shielding, indicating non‐uniform stress distribution between the implant and the adjacent bone due to differences in stiffness. Specifically, the disparity in stiffness between the implant (E = 110 GPa) and the healing bone (E = 0.16 GPa) led to a higher stress concentration on the implant body compared to the adjacent bone.

In cortical bone, Model B exhibited a maximum von Mises stress of 72 MPa, while Model A showed a stress of 64 MPa. Within healing bone, the stress distribution for Model B ranged from 1.5 MPa to 12 MPa along the implant body. In contrast, Model A displayed a lower stress distribution between 1.3 MPa and 9 MPa.

**FIGURE 6:**
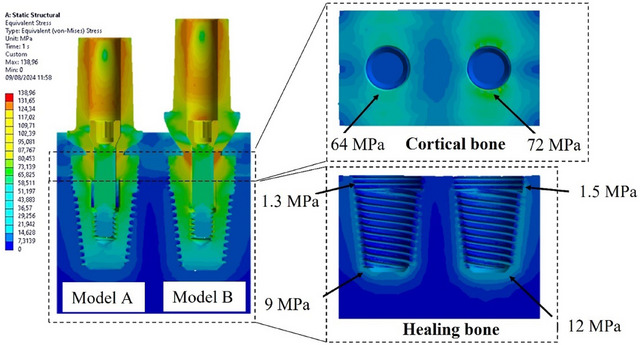
Stress distribution on the bone.

These findings suggested that the abutment profile significantly impacts stress distribution within the bone. Specifically, the implant configuration utilizing abutment Model B resulted in higher but more evenly distributed stresses along the implant profile (Figure 6).

### Stress distribution on soft tissues

**FIGURE 7:**
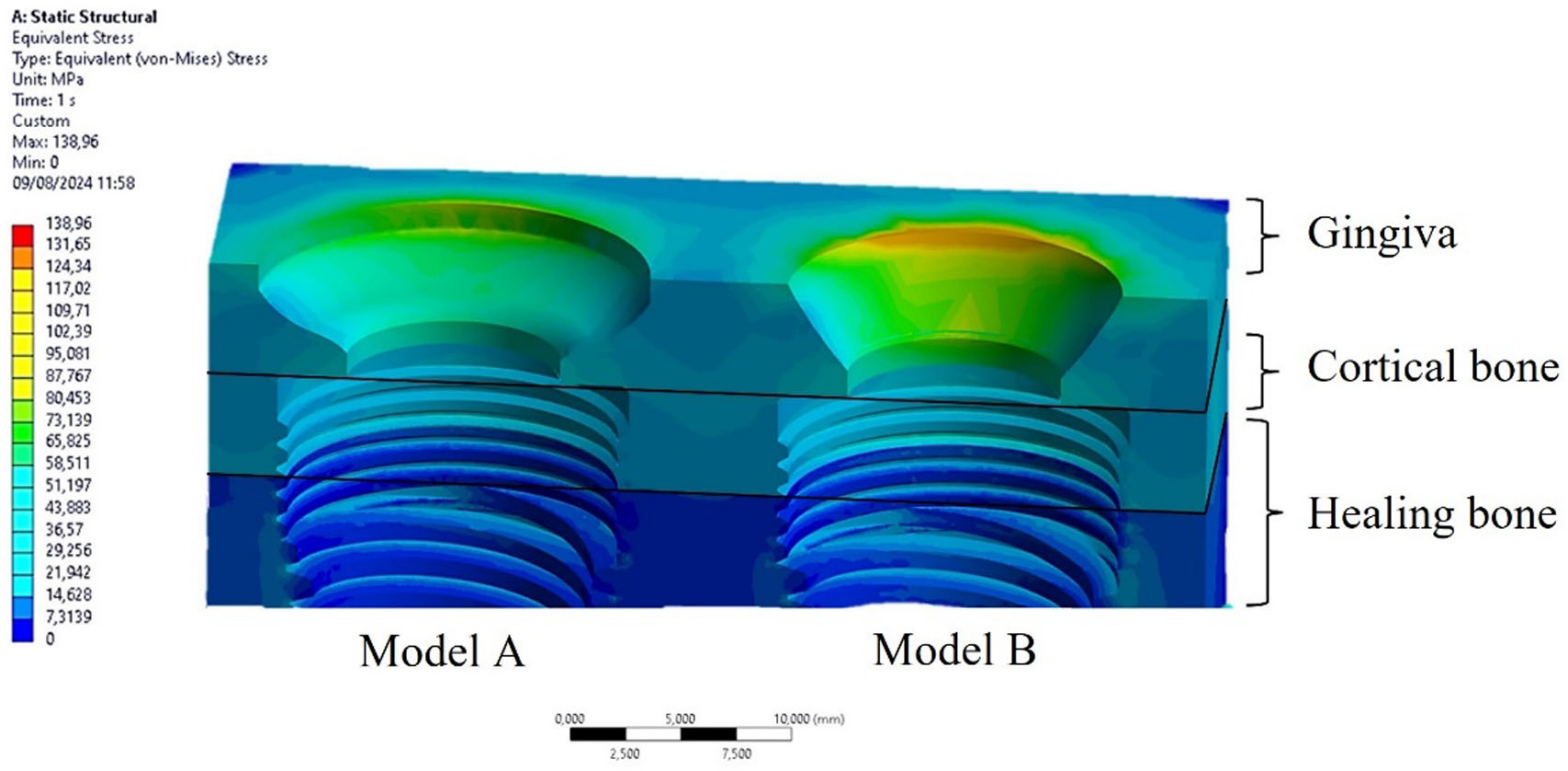
Von Mises stress distribution on the gingiva, cortical bone, and healing bone.

The gingiva is a soft tissue with a remarkably low stiffness of 0.0197 GPa. Consequently, it experiences higher levels of stress compared to the cortical bone. In cortical bone, the stress was observed to vary between 64 MPa and 72 MPa, as outlined in section 3.2. In comparison, for gingival tissues, the stress ranged from 75 MPa in Model A to 126 MPa in Model B. This data indicated that gingiva experiences the highest levels of stress among the assessed tissues (Figure 7).

## DISCUSSION

The objective of this study was to evaluate the impact of mechanical stress on peri‐implant tissues in post‐extraction conditions, encompassing both hard and soft tissues, by employing a PHI with two distinct transmucosal abutment designs: concave (Model A) and divergent (Model B). The findings suggested that implant Model B elicited greater stress on both the gingiva and the bone. Consequently, the initial null hypothesis stating that post‐extraction bone behaves similarly around both abutment models was rejected.

The original implant treatment protocol, as proposed by Brånemark, traditionally involved a healing period after tooth extraction, during which the patient remained edentulous before the placement of implants.^59^ However, post‐extraction resorption and remodeling of the alveolar bone can lead to insufficient bone volume for implant placement.^60^ Immediate implant placement, performed at the time of tooth extraction, takes advantage of the osteogenic responses initiated after extraction, facilitating improved healing and significantly reducing overall healing time.^60^ This approach offers several benefits, including reduced treatment duration, preservation of alveolar ridge contour, and the opportunity for optimal implant positioning to enhance both aesthetics and biomechanics.^62^, ^63^

However, the long‐term success of dental implants depends on a multitude of interrelated factors. The health of the surrounding tissues is paramount, as the alveolar bone needs to withstand masticatory forces while the overlying soft tissue must shield the bone from the intraoral environment.^31^, ^64^ Maintenance of a stable and immobile soft tissue seal around the implants is crucial to prevent damage.^65^ Furthermore, the implant connection plays a pivotal role, with internal connections aiding in maintaining bone integrity through adequate stimulation without compromising the soft tissue seal.^65^, ^66^ The shape and profile of the abutment can also impact MBL and soft tissue sealing.^68^

In dentistry, the FEM is employed to simulate and analyze the behavior of dental implants, prostheses, bone tissue, and other components of the stomatognathic system under various occlusal loads. This method aids in evaluating the stress distribution around dental implants and surrounding bone structures.^69^, ^70^ Through this approach, prosthetic structures can be designed and optimized to enhance peri‐implant tissue health and improve long‐term durability. For instance, a finite element study by Hsieh et al.^71^ revealed that the implant model with a convergently shaped crestal tract induced higher stress concentration around the cortical bone, particularly under oblique loading conditions, thereby fostering the process of osseointegration and sustaining implant stability.

The present study utilized FEA to explore the mechanical behavior of a PHI when applied with the primary healing technique within bone tissue.^72^ The investigation focused on an implant featuring a conometric connection linking the abutment and the implant. This particular configuration offers noteworthy benefits within the realm of dentistry and dental implants.^73^, ^74^ The conometric connection provides enhanced mechanical stability between the implant and abutment, thereby decreasing the likelihood of undesired movement and unscrewing.^75^, ^76^ Moreover, it facilitates uniform stress distribution along the implant interface, which aids in enhancing implant biomechanics and reducing stress concentration within the peri‐implant region. Furthermore, the tapered connection serves to mitigate the formation of micro‐gaps between the implant and abutment, thus reducing the risk of bacterial infiltration and peri‐implant bone loss, as evidenced by an in vitro study.^77^ In addition, differing stress distributions on the peri‐implant tissues were observed between two abutment designs (Model A and Model B). Notably, in configuration A, characterized by a transmucosal tract with a pronounced convergent zone, lower stress transmission to the hard tissue was evident in comparison to configuration B. This outcome was corroborated by a systematic review and meta‐analysis by Valente et al.,^30^ which demonstrated that implants featuring convergent or concave transmucosal profiles exhibited superior results in terms of MBL when compared to implants with divergent or parallel transmucosal profiles or those with less pronounced curvatures. By incorporating the gingiva into the 3D model, this study has contributed to a deeper comprehension of the mechanical behavior of diverse peri‐implant tissues. Understanding the state of soft tissue loading is essential for grasping how the components of the implant system (namely, the abutment and the implant) can influence soft tissue health from both clinical and biomechanical perspectives. As indicated by the research undertaken by Rodriguez et al.,^78^ soft tissues exhibit varying tensile and compressive strengths, ranging from 4 to 7 MPa. Therefore, if the loading on these tissues surpasses these thresholds, their biomechanical integrity may be jeopardized. It is important to note, however, that there is a shortage of FEA studies in the literature incorporating soft tissues into simulations.^78^, ^79^, ^80^

It is important to note that the stress findings reported for bone in this study exceeded those documented in the literature,^81^, ^82^, ^83^, ^84^, ^85^, ^86^ as the characteristics of post‐extraction phase bone differ from those of the mature bone occurring after 4–6 months. However, the modeling of anatomy and bone quality in this study is subjected to limitations as it only considered cortical thickness and Young's modulus of cortical and post‐extraction bone. On the other hand, there was a notable advancement in automating the digital chain, particularly in the segmentation of scanned images.^87^ This enables the swift and nearly automatic generation of a digital model that accounts for the patient's actual anatomy and bone density. Consequently, mechanical criteria can be integrated into the planning phase of implant placement by dental surgeons. The parametric model also incorporates adhesive contacts, specifically at the bone‐to‐implant interface, yet it does not allow for modeling different levels of osseointegration. Various techniques have been explored in the literature to address this limitation, such as adjusting the coefficient of friction or manipulating the number of nodes.^88^ It is important to note that bone behaves differently in different directions, and the use of the isotropy hypothesis primarily aims to reduce computational time by focusing on other aspects. Further studies should encompass this aspect and consider how bone behaves differently in different anatomical zones and directions. Another limitation pertains to the simulation of gingival behavior within this study. The gingiva demonstrates hyperelastic characteristics, allowing it to sustain considerable deformations without failure. The simplified elastic model utilized in this study may not accurately capture these deformations, potentially leading to erroneous simulation outcomes. Furthermore, the gingiva possesses anisotropic properties, indicating that its mechanical attributes may vary depending on the direction of applied stress. Basic elastic models are insufficient in representing this anisotropy, while hyperelastic models can be adjusted to account for such variations. Therefore, forthcoming studies that evaluate the impact of hyperelasticity on results may significantly enhance the field of soft tissue modeling. Additionally, it is crucial to address the use of dynamic analysis rather than static analysis. This limitation aligns with the unclear dynamic cycle of mastication, and future studies should implement load variation over time for a more comprehensive analysis.

The findings of this study offer valuable insights into the influence of abutment design on the stability of implants subjected to immediate loading, as well as the health of peri‐implant tissues. By simulating a prosthetic abutment in conjunction with a PHI implant, this research enables the evaluation of the forces and stresses encountered during the early stages of healing. Such an analysis allows for the identification of potential criticalities prior to intervention. Should the results indicate that immediate loading is both safe and effective, clinicians will be empowered to adopt this practice with increased confidence, thereby decreasing waiting times for patients. Additionally, the findings can inform the design of prosthetic abutments that enhance force distribution and mitigate stress, particularly in instances of compromised bone quality. This approach could potentially lower the risk of complications and enhance the longevity of the implant.

## CONCLUSIONS

The findings of this study demonstrated the favorable biomechanical performance of PHIs positioned in post‐extraction sites. By employing the FEA to simulate bone behavior at the post‐extraction site, the study examined stress distribution in the hard and soft tissues. The results also underscored the significant influence of abutment profile shape on the distribution of occlusal loads on prosthetic components and peri‐implant tissues. Utilizing the FEM approach enabled the design and optimization of implant shapes based on occlusal loads and anatomical locations to promote soft and hard tissue stimulation and mitigate the risk of implant failure. Further experimental testing is warranted to corroborate the present hypotheses and findings.

## CONFLICT OF INTEREST STATEMENT

The authors declare no conflicts of interest.
